# PCSK9 Plasma Concentrations Are Independent of GFR and Do Not Predict Cardiovascular Events in Patients with Decreased GFR

**DOI:** 10.1371/journal.pone.0146920

**Published:** 2016-01-22

**Authors:** Kyrill S. Rogacev, Gunnar H. Heine, Günther Silbernagel, Marcus E. Kleber, Sarah Seiler, Insa Emrich, Simone Lennartz, Christian Werner, Adam M. Zawada, Danilo Fliser, Michael Böhm, Winfried März, Hubert Scharnagl, Ulrich Laufs

**Affiliations:** 1 Department of Internal Medicine III, Cardiology, Angiology and Intensive Care Medicine, Saarland University Medical Center, Homburg, Germany; 2 Department of Internal Medicine IV, Nephrology and Hypertension, Saarland University Medical Center, Homburg, Germany; 3 Division of Angiology, Swiss Cardiovascular Center, Bern University Hospital, Bern, Switzerland; 4 Vth Department of Medicine (Nephrology, Hypertensiology, Endocrinology, Diabetology, Rheumatology), Medical Faculty Mannheim, Heidelberg University, Mannheim, Germany; 5 Synlab Academy, Synlab Services GmbH, Mannheim and Augsburg, Germany; 6 Clinical Institute of Medical and Chemical Laboratory Diagnostics, Medical University of Graz, Graz, Austria; KRH Robert Koch Klinikum Gehrden, GERMANY

## Abstract

**Background:**

Impaired renal function causes dyslipidemia that contributes to elevated cardiovascular risk in patients with chronic kidney disease (CKD). The proprotein convertase subtilisin/kexin type 9 (PCSK9) is a regulator of the LDL receptor and plasma cholesterol concentrations. Its relationship to kidney function and cardiovascular events in patients with reduced glomerular filtration rate (GFR) has not been explored.

**Methods:**

Lipid parameters including PCSK9 were measured in two independent cohorts. CARE FOR HOMe (Cardiovascular and Renal Outcome in CKD 2–4 Patients—The Forth Homburg evaluation) enrolled 443 patients with reduced GFR (between 90 and 15 ml/min/1.73 m^2^) referred for nephrological care that were prospectively followed for the occurrence of a composite cardiovascular endpoint. As a replication cohort, PCSK9 was quantitated in 1450 patients with GFR between 90 and 15 ml/min/1.73 m^2^ enrolled in the Ludwigshafen Risk and Cardiovascular Health Study (LURIC) that were prospectively followed for cardiovascular deaths.

**Results:**

PCSK9 concentrations did not correlate with baseline GFR (CARE FOR HOMe: r = -0.034; p = 0.479; LURIC: r = -0.017; p = 0.512). 91 patients in CARE FOR HOMe and 335 patients in LURIC reached an endpoint during a median follow-up of 3.0 [1.8–4.1] years and 10.0 [7.3–10.6] years, respectively. Kaplan-Meier analyses showed that PCSK9 concentrations did not predict cardiovascular events in either cohort [CARE FOR HOMe (p = 0.622); LURIC (p = 0.729)]. Sensitivity analyses according to statin intake yielded similar results.

**Conclusion:**

In two well characterized independent cohort studies, PCSK9 plasma levels did not correlate with kidney function. Furthermore, PCSK9 plasma concentrations were not associated with cardiovascular events in patients with reduced renal function.

## Introduction

Patients with decreased glomerular filtration rate (GFR) are at high risk for cardiovascular (CV) events [1]. Their elevated CV risk is caused by a complex interplay of non-traditional risk factors, such as inflammation [2], dysregulated calcium-phosphate metabolism [3] and traditional risk factors such as dyslipidemia and hypertension [4]. Dyslipidemia in patients with impaired renal function is characterized by qualitative changes in cholesterol homeostasis [5] and reverse cholesterol transport [6], and quantitative changes of lipid parameters [7]. Progressive kidney function loss is accompanied by a rise of triglycerides and VLDL-cholesterol (VLDL-C); at the same time total cholesterol, HDL-cholesterol (HDL-C) and LDL-cholesterol (LDL-C) decrease [7]. Specifically, baseline mean LDL-C in the large statin trials in chronic kidney disease (CKD) patients were in the relatively low range of 100–120 mg/dl [8–10]. The underlying mechanisms of CKD associated dyslipidemia and especially the reason for low LDL-C serum concentrations are not fully understood.

Hepatic uptake of LDL-C by the LDL receptor is the major route of LDL clearance from the blood. In the last decade a new, central regulator of LDL receptor expression, namely proprotein convertase subtilisin/kexin type 9 (PCSK9), has been identified (as reviewed in [11]). PCSK9 facilitates LDL receptor degradation and inhibits the receptor’s recycling to the membrane. Gain-of-function mutations of PCSK9 have been linked with elevated LDL-C whereas loss-of-function mutations are tied to low LDL-C and reduced CV risk. Thus, PCSK9 has become a promising drug target in CV medicine with several drug development programs currently underway. As evidenced by the statin trials in hemodialysis patients (4D and AURORA) [9,10] and other trials aiming to improve CV prognosis [12], patients with chronic kidney disease differ from other individuals with high CV risk. The reasons for this difference are not fully understood. In this respect it is unknown whether kidney function affects PCSK9 levels. In addition, it is not known whether PCSK9 levels correlate with CV risk in patients with decreased GFR.

In the current study we therefore aimed to analyze the relationship between kidney function and PCSK9. Furthermore, we asked whether PCSK9 predicts CV risk in patients with decreased glomerular filtration rate. The results of the CARE FOR HOMe study (Cardiovascular and Renal Outcome in CKD 2–4 Patients—The Forth Homburg evaluation) were confirmed in the Ludwigshafen Risk and Cardiovascular Health Study (LURIC).

## Materials and Methods

PCSK9 plasma concentrations were assessed in the CARE FOR HOMe (Cardiovascular and Renal Outcome in CKD 2–4 Patients—The Forth Homburg evaluation) study. The results were confirmed in the LURIC study (Ludwigshafen Risk and Cardiovascular Health Study). Both studies were conducted in accordance with the Declaration of Helsinki.

### Study description–CARE FOR HOMe

The CARE FOR HOMe study is an ongoing, prospective cohort study in CKD patients which aims to characterize cardio-renal interactions [13–15]. Recruitment started in September 2008 with an average recruitment rate of 100 patients per year. Study participants were referred for nephrological care to the outpatient clinic of the Department of Internal Medicine IV of Saarland University Medical Centre. Patients with CKD GFR categories 2–4, corresponding to a glomerular filtration rate (GFR) between 90 and 15 ml/min/1.73 m^2^, were invited to participate. Unstable clinical status (active malignancy, systemic infection), acute kidney injury and intake of immunosuppressant drugs were defined as exclusion criteria.

Information on co-morbidity was gathered by a standardized questionnaire and confirmed by chart review. Pre-existent cardiovascular disease was defined as a history of myocardial infarction, coronary artery angioplasty / stenting / bypass surgery, major stroke, carotid endarterectomy / stenting, nontraumatic lower extremity amputation, or lower limb artery angioplasty / stenting / bypass surgery. Patients with self-reported or physician-reported diabetes mellitus, with a fasting blood glucose level of ≥126 mg/dl and / or with current use of hypoglycemic medication, were defined as diabetic. Patients were categorized as active smokers if they were current smokers or had stopped smoking < 1 month before entry into the study.

Blood pressure was measured after 5 mins of rest with an automated blood pressure recording apparatus (GE Carescape DINAMAP V100; GE Healthcare). Body mass index (BMI) was calculated as weight (kg)/[height (m)]^2^.

The local Ethics Committee (Ethikkommision der Aerztekammer des Saarlandes for CARE FOR HOMe and Ethikkommision der Aerztekammer von Rheinland-Pfalz) approved the study and all participants gave their written informed consent.

### Laboratory measurements in CARE FOR HOMe

In CARE FOR HOMe participants, we obtained blood samples after an overnight fast. All routine laboratory measurements in CARE FOR Home were performed at the core facility for Clinical Chemistry and Laboratory Medicine of Saarland University Medical Centre. Fasting PCSK9 concentrations were measured by enzyme-linked immunosorbent assay using the Circulex Human PCSK9 ELISA Kit (CY-8079, CycLex, Japan) in diluted (1:100) fasting plasma samples according to the manufacturer’s instructions. A seven-point standard curve (range 0.16 to 10 ng/ml) was created using lyophilized human PCSK9.

### End-points in CARE FOR HOMe

All CARE FOR HOMe patients were invited annually for follow-up examinations. The composite CV end-point was defined as the first occurrence of any of the following: acute myocardial infarction; surgical or interventional coronary / cerebrovascular / peripheral-arterial revascularization; stroke with symptoms ≥ 24 hours, amputation above the ankle; or death of any cause. Myocardial infarction was defined according to the Joint ESC/ACCF/AHA/WHF task force consensus criteria.[16] Outcome adjudication was performed by two physicians blinded to baseline PCSK9 levels. In case of disagreement, a third physician gave an assessment blinded to those of the previous two doctors. For all events confirmatory medical documentation was obtained. This outcome adjudication process has been described previously.[17]

### Study description–LURIC

Methods and the study population of LURIC have been described in detail earlier.[18] The LURIC study included 3,316 patients referred for coronary angiography between 1997 and 2000 at a tertiary care centre in south-western Germany. Clinical indications for angiography were chest pain or a positive non-invasive stress test suggestive of myocardial ischemia. To limit clinical heterogeneity, individuals suffering from acute illnesses other than acute coronary syndrome, chronic non-cardiac diseases and a history of malignancy within the five past years were excluded. Study participants were followed-up for a median of 10 years. 1462 LURIC participants had PCSK9 measurements and a GFR between 90 and 15 ml/min/1.73m^2^ estimated by the MDRD formula corresponding to the GFR inclusion criterion of CARE FOR HOMe. Of those, 1450 had complete data on vital status and causes of death.

### Laboratory measurements in LURIC

Fasting blood samples were collected in the early morning with patients in the supine position. Routine laboratory parameters were measured on a daily basis as previously described[18]. The lipoproteins were separated using a combined ultracentrifugation–precipitation method (β-quantification). Cholesterol and triglycerides were measured with enzymatic reagents from WAKO (Neuss, Germany) on a WAKO 30 R analyser. Apolipoproteins A-I, and B were measured by turbidimetry (Rolf-Greiner Biochemica, Flacht, Germany). Creatinine was measured with the Jaffé method on a Hitachi 717 analyzer (Roche Diagnostics, Mannheim, Germany). The CRP concentrations were determined using immunonephelometry (N High Sensitivity CRP, Dade Behring, Marburg, Germany). Total PCSK9 was measured using the Quantikine Human PCSK9 sandwich immunoassay (R&D, Minneapolis, MN, USA).

### Endpoints in LURIC

Information on vital status was obtained from local registries No patient was lost to follow-up. Causes of death were determined using death certificates. Patients were classified into those who died of cardiovascular versus those who died of non-cardiovascular causes. Cardiovascular death included sudden death, fatal myocardial infarction, death due to congestive heart failure, death immediately after intervention to treat coronary heart disease (CHD), fatal stroke, and other causes of death due to CHD. In LURIC STEMI was diagnosed if typical ECG changes were present along with prolonged chest pain, refractory to sublingual nitrates and / or enzyme or troponin T elevations (>0.1 mg/L). Non-ST elevation MI (NSTEMI) was diagnosed, if symptoms and/or troponin T criteria were met without STEMI ECG criteria. This classification was performed independently by two experienced clinicians who were blinded to the study participants. In the case of disagreement or uncertainty, a decision was made by one of the LURIC study principal investigators (W.M.). The adjudication process has been described previously.[19]

### Statistical analysis applied in CARE FOR HOMe and LURIC

Data management and statistical analysis were performed using PASW Statistics 22 (SPSS, Inc., Chicago, Illinois). Two-sided p values < 0.05 were considered significant.

For clinical data, categorical variables are presented as percentages of patients and were compared using chi-square or Fisher’s exact tests, as appropriate. Continuous data are expressed as median and interquartile range and compared using Mann-Whitney U test for two independent samples or Kruskal-Wallis test for more than two independent samples, followed by Jonckheere-Terpstra test for trend. The associations between continuous variables were assessed using Spearman correlation testing. Patients were divided into 3 equally sized groups (tertiles) according to their levels of PCSK9. Kaplan-Meier survival curves were used to compare event-free survival (i.e., time until first occurrence of the composite endpoint) between groups. The log-rank test was used to test the hypothesis that at least one of the survival curves differs from the others. In CARE FOR HOMe Cox proportional hazard models were calculated to analyze the relationship of lipid parameters (PCSK9, total cholesterol, Apo A-I, Apo B) with event-free survival after adjustment for GFR, age, mean BP, gender, prevalence of CV disease, diabetes mellitus and statin intake; in LURIC Cox proportional models were built with baseline variables differing between those participants with an event compared to those without an event (gender, prevalent cardiovascular disease, smoking, diabetes mellitus, age, BMI, systolic blood pressure, GFR, apo A-I and hsCRP).

## Results

### PCSK9 concentrations and baseline characteristics

Baseline characteristics of CARE FOR HOMe participants according to tertiles of PCSK9 are summarized in **Table 1**.

**Table 1:** Baseline characteristics of CARE FOR HOMe participants stratified by PCSK9 tertiles.

	Total Cohort	1^st^ Tertile	2^nd^ Tertile	3^rd^ Tertile	p-value
	N = 443	N = 147	N = 149	N = 147	
**Gender (men)**	265 (60%)	101 (69%)	86 (58%)	78 (53%)	**0.019**
**Prevalent CVD (yes)**	135 (30%)	42 (29%)	42 (28%)	51 (35%)	0.396
**Smoking (yes)**	44 (10%)	15 (10%)	13 (9%)	16 (11%)	0.817
**Diabetes (yes)**	168 (38%)	52 (35%)	57 (38%)	59 (40%)	0.698
**Age (years)**	67.7 [57.0–74.3]	67.7 [55.7–74.4]	59.6 [69.8–74.5]	65.1 [55.9–73.9]	0.266
**BMI (kg/m**^**2**^**)**	29.8 [26.1–33.1]	29.2 [25.3–32.7]	29.7 [25.7–33.4]	30.5 [27.1–33.4]	***0*.*035***
**Mean BP (mmHg)**	108 [99–118]	108 [101–119]	109 [98–120]	108 [100–116]	0.756
**GFR (ml/min/1.73 m**^**2**^**)**	46.1 [33.4–56.7]	46.7 [35.3–57.4]	46.2 [33.2–57.5]	43.4 [32.2–54.6]	0.410
**UAE (mg/g creatinine)**	35 [8–193]	34 [9–149]	32 [7–181]	41 [8–254]	0.888
**PCSK9 (ng/ml)**	343 [270–413]	246 [215–270]	343 [323–366]	471 [413–534]	***<0*.*001***
**Total Cholesterol (mg/dl)**	188 [162–219]	185 [162–209]	189 [159–224]	195 [166–221]	0.243
**HDL Cholesterol (mg/dl)**	48 [39–61]	47 [38–61]	49 [41–64]	48 [40–59]	0.620
**LDL Cholesterol (mg/dl)**	114 [90–138]	114 [89–135]	116 [93–141]	113 [89–140]	0.674
**Triglycerides (mg/dl)**	135 [97 - 190]	116 [88 - 164]	137 [106 - 200]	156 [107 - 232]	***< 0*.*001***
**Apo A-I (mg/dl)**	161 [142–185]	156 [137–182]	161 [144–186]	165 [148–188]	***0*.*049***
**Apo B (mg/dl)**	97 [82–115]	94 [79–110]	98 [80–114]	100 [86–117]	0.118
**hsCRP (mg/l)**	2.7 [1.2 – 5.3]	2.7 [1.1 - 5.4]	2.6 [1.2 - 5.1]	3.0 [1.2 – 5.6]	0.795
**Statin (yes)**	216 (49 %)	48 (33%)	79 (53%)	89 (61%)	**< 0.001**

**CVD:** cardiovascular disease; **BMI:** body mass index; **BP:** blood pressure; **GFR:** glomerular filtration rate; **UAE:** urinary albumin excretion; **PCSK9:** proprotein convertase subtilisin/kexin type 9; **Apo A-I, B:** apolipoprotein A, B; **hsCRP:** high-sensitivity C-reactive protein. Data are presented as numbers (percentages) or median and interquartile range as appropriate.

**Fig 1:**
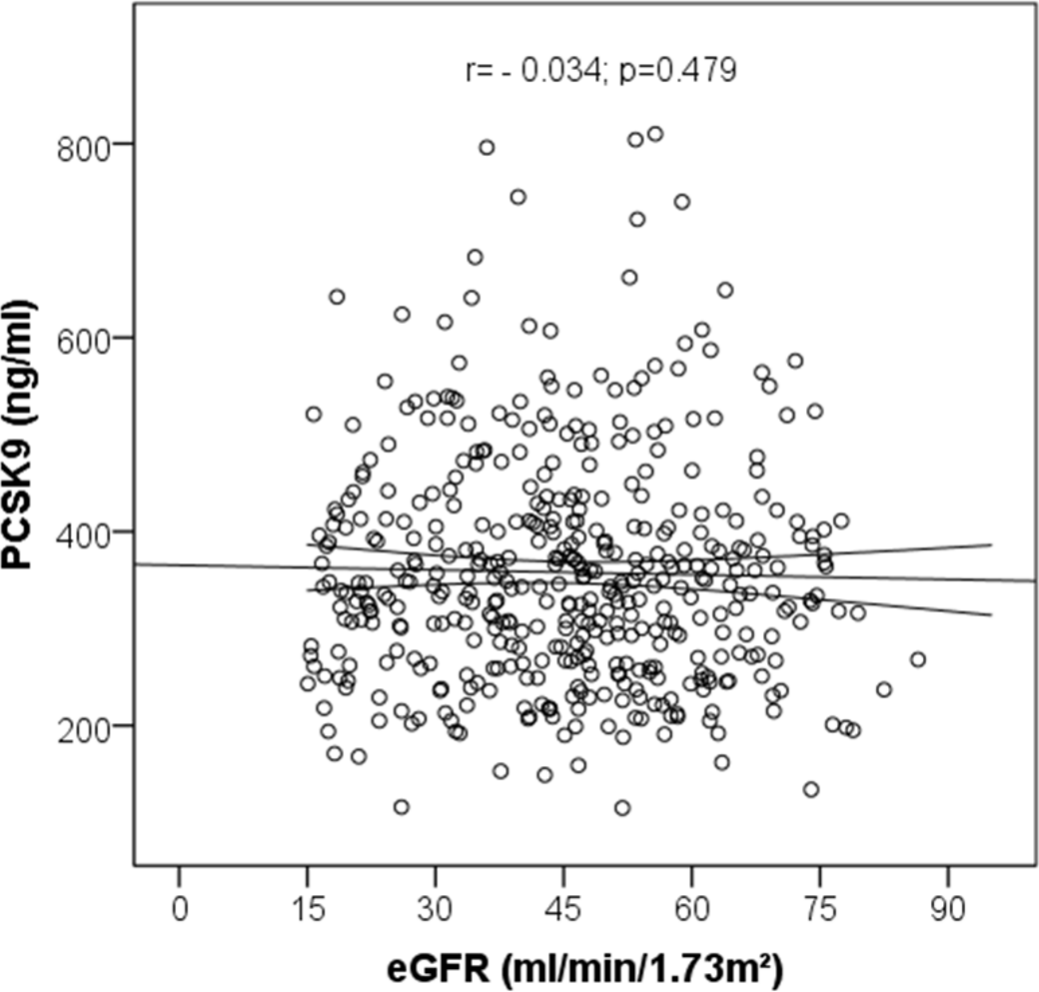
Correlation between PCSK9 and estimated glomerular filtration rate (GFR) assessed by Spearman test.

In the entire cohort, PCSK9 levels weakly correlated with BMI (r = 0.125; p = 0.008), total cholesterol (r = 0.102; p = 0.031), triglycerides (r = 0.190; p < 0.001), Apo A-I (r = 0.149; p = 0.002) and Apo B (r = 0.114; p < 0.018), but not with LDL cholesterol (r = 0.051; p = 0.287) or GFR (r = -0.034; p = 0.479; **Fig 1**).

### Characteristics according to statin intake across categories of kidney function

**Fig 2:**
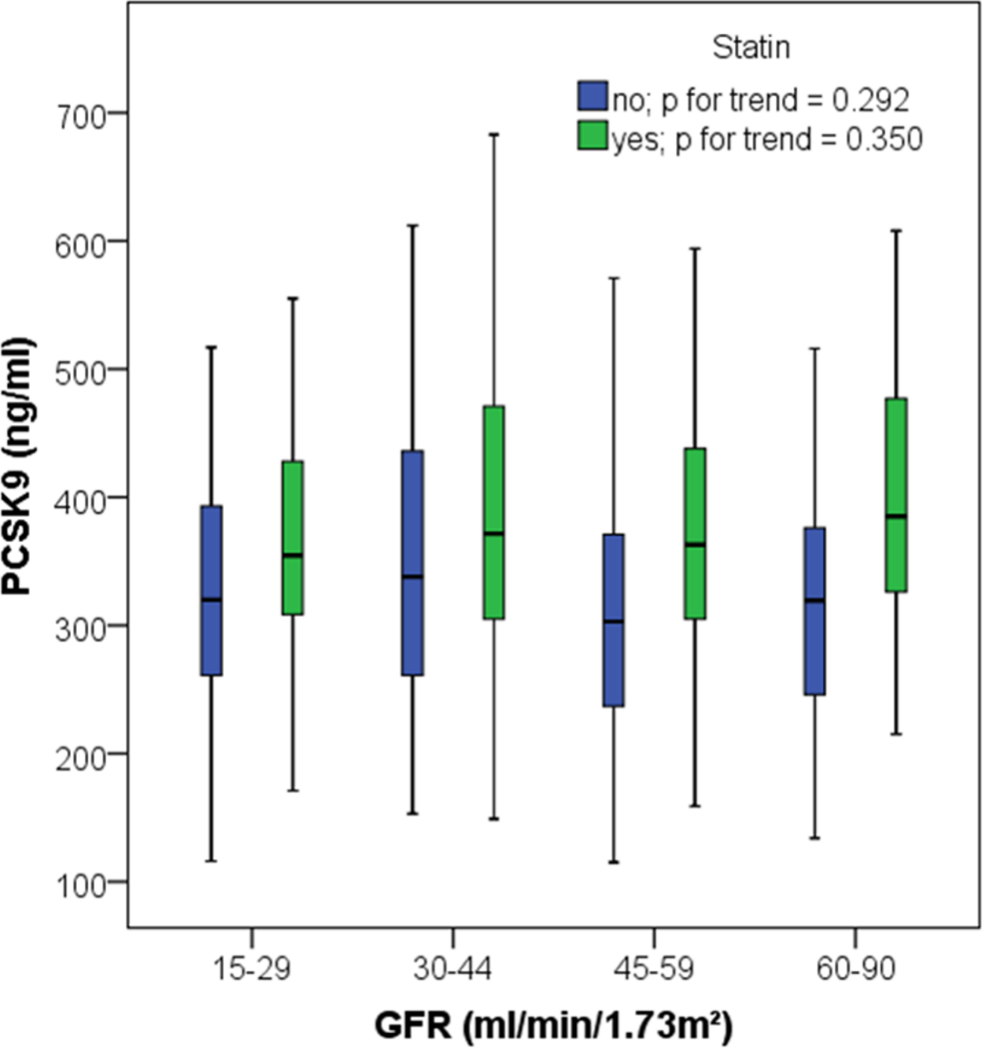
Association of PCSK9 with strata of GFR in patients taking statins and patients not on statin medication; Jonckheere Terpstra test for comparison among statin users, respectively statin non-users, across GFR strata.

216 (49%) out of 443 patients were taking statins. Patients were stratified according to statin intake across GFR categories. In statin users vs. non-statin users, the proportion of patients with prevalent CV disease was higher (100/216 vs 35/227; p<0.001) whereas no difference was found regarding gender distribution (men: 131/216 vs 134/227; p = 0.771), diabetic status (diabetics: 91/216 vs 77/227; p = 0.079) and smoking status (20/216 vs 24/227; p = 0.751). Statin users in comparison to statin non-users were significantly older (67.9 ± 10.2 vs 62.4 ± 13.8 yrs; p<0.001) and had significantly lower GFR (43.6 ± 15.0 vs 46.9 ± 16.7 ml/min/1.73 m^2^; p = 0.031). The other characteristics of the two subgroups were similar. PCSK9 levels did not differ across GFR categories in statin users (p for trend = 0.350) and statin non-users (p for trend = 0.292) as shown in **Fig 2**.

However, within each GFR category statin users had higher PCSK9 concentrations compared to statin non-users (GFR 15–29 ml/min/1.73 m^2^: p = 0.031; GFR 30–44 ml/min/1.73 m^2^: p = 0.170; GFR 45–59 ml/min/1.73 m^2^: p <0.001; GFR 60–90 ml/min/1.73 m^2^: p = 0.001).

### Clinical follow-up in CARE FOR HOMe

**Fig 3:**
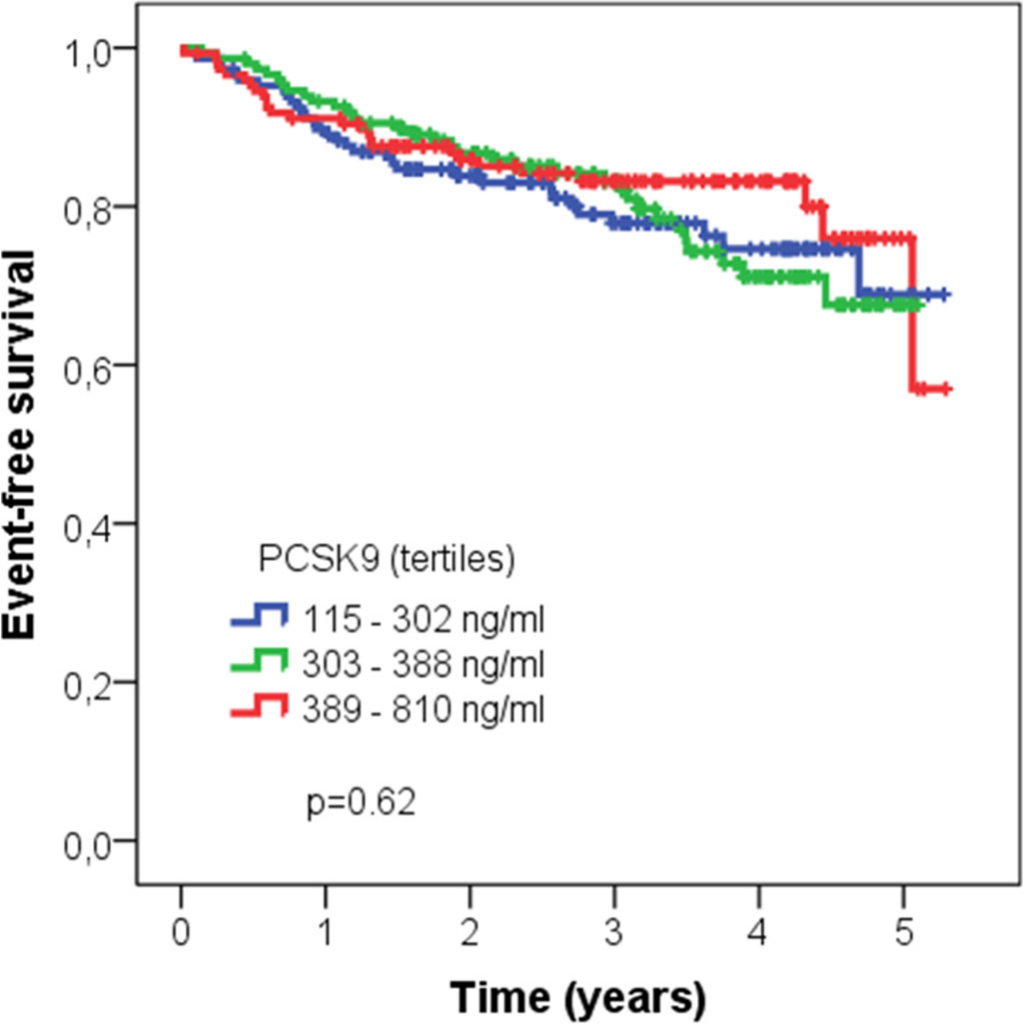
Association of tertiles of PCSK9 with cardiovascular outcome; Kaplan-Meier analysis followed by log-rank test.

Median follow-up time was 3.0 [1.8–4.1] years, during which 91 patients reached the primary end-point. Characteristics of patients according event status are summarized in **Table 2**. No patient was lost to follow-up. We analyzed the relationship between PCSK9 and CV outcome by Kaplan-Meier analysis, demonstrating no significant association between tertiles of PCSK9 and CV outcome (**Fig 3**).

**Table 2:** Baseline characteristics stratified by event status.

	Total Cohort	No Event	Event	p-value
	N = 443	N = 352	N = 91	
**Gender (men)**	265 (60%)	201 (57%)	64 (70%)	**0.023**
**Prevalent CVD (yes)**	135 (30%)	78 (22%)	57 (63%)	**<0.001**
**Smoking (yes)**	44 (10%)	33 (9%)	11 (12%)	0.435
**Diabetes (yes)**	168 (38%)	122 (35%)	46 (51%)	**0.007**
**Age (years)**	67.7 [57.0–74.3]	65.5 [55.2–73.7]	72.7 [66.4–77.3]	**<0.001**
**BMI (kg/m**^**2**^**)**	29.8 [26.1–33.1]	30.0 [26.2–33.4]	28.8 [26.0–32.6]	0.143
**Mean BP (mmHg)**	108 [99–118]	109 [101–119]	104 [91–115]	**0.002**
**GFR (ml/min/1.73 m**^**2**^**)**	46.1 [33.4–56.7]	47.9 [36.8–58.7]	34.3 [26.0–45.2]	**<0.001**
**UAE (mg/g creatinine)**	35 [8–193]	27 [7–157]	80 [22–315]	**0.001**
**PCSK9 (ng/ml)**	343 [270–413]	349 [269–421]	330 [272–409]	0.316
**Total Cholesterol (mg/dl)**	188 [162–219]	195 [167–223]	171 [150–195]	**<0.001**
**HDL Cholesterol (mg/dl)**	48 [39–61]	49 [41–63]	44 [36–53]	**0.001**
**LDL Cholesterol (mg/dl)**	114 [90–138]	116 [93–140]	100 [82–123]	**0.001**
**Triglycerides (mg/dl)**	135 [97–190]	135 [96–195]	132 [97–183]	0.632
**Apo A-I (mg/dl)**	161 [142–185]	165 [146–189]	148 [132–165]	**<0.001**
**Apo B (mg/dl)**	97 [82–115]	99 [83–116]	92 [78–105]	**0.008**
**hsCRP (mg/l)**	2.7 [1.2–5.3]	2.6 [1.1–4.8]	3.9 [1.6–9.6]	**0.003**
**Statin (yes)**	216 (49%)	161 (46%)	55 (60%)	**0.014**

**CVD:** cardiovascular disease; **BMI:** body mass index; **BP:** blood pressure; **GFR:** glomerular filtration rate; **UAE:** urinary albumin excretion; **PCSK9:**proprotein convertase subtilisin/kexin type 9; **Apo A-I, B:**apolipoprotein A, B;**hsCRP:** high-sensitivity C-reactive protein. Data are presented as numbers (percentages) or median and interquartile range as appropriate.

Kaplan-Meier analyses of the subgroups with and without statin treatment were performed separately; in these sensitivity analyses consistently with the overall cohort no association between tertiles of PCSK9 (statin users: p = 0.367; statin non-users: p = 0.834) and outcome was seen.

Finally, in multivariate analyses we adjusted for confounders, including lipid parameters (log PCSK9, total cholesterol, Apo A-I, Apo B), GFR, traditional CV risk factors (age, sex, prevalent CV disease) and statin intake; after full adjustment only GFR, age, prevalent CV disease and diabetes mellitus were independent predictors of CV outcome (**Table 3**).

**Table 3:** Adjusted Cox regression analysis (different models) for cardiovascular events in CARE FOR HOMe.

	HR Mod1	95% CI	p-value	HR Mod2	95% CI	p-value	HR Mod3	95% CI	p-value
**GFR (ml/min/1.73 m**^2^**)**	0.974	(0.959, 0.989)	0.001	0.972	(0.957, 0.987)	<0.001	0.972	(0.957, 0.987)	<0.001
**Age (years)**	1.045	(1.022, 1.069)	<0.001	1.031	(1.007, 1,056)	0.012	1.031	(1.007, 1.056)	0.012
**Log PCSK9**	0.570	(0.121, 2.685)	0.477	0.330	(0.062, 1.751)	0.193	0.333	(0.060, 1.832)	0.206
**Total-C (mg/dl)**	0.992	(0.979, 1.004)	0.176	0.994	(0.982, 1.006)	0.298	0.994	(0.982, 1.006)	0.307
**Apo A-I (mg/dl)**	0.990	(0.980, 1.000)	0.041	0.996	(0.987, 1.006)	0.470	0.997	(0.987, 1.006)	0.483
**Apo B (mg/dl)**	1.004	(0.984, 1.024)	0.729	1.003	(0.984, 1.022)	0.748	1.003	(0.984, 1.022)	0.745
**Mean BP (mmHg)**	0.994	(0.979, 1.009)	0.411	0.986	(0.971, 1.001)	0.072	0.986	(0.971, 1.001)	0.072
**Gender (male)**				1.310	(0.811, 2.115)	0.269	1.311	(0.811, 2.117)	0.269
**Prevalent CVD (yes)**				3.069	(1.934, 4.870)	<0.001	3.078	(1.910, 4.961)	<0.001
**Diabetes mellitus (yes)**				1.616	(1.053, 2.479)	0.028	1.617	(1.053, 2.485)	0.028
**Statin (yes)**							0.988	(0.606, 1.609)	0.960

**GFR:** glomerular filtration rate; **PCSK9**: proprotein convertase subtilisin/kexin type 9; **Total-C**: total cholesterol; **ApoA- I, B:** apoliporotein A, B; **Mean BP**: mean blood pressure; **CVD**: cardiovascular disease. Model 1 includes GFR, age, PCSK9, total cholesterol, Apo A-I, Apo B and mean BP. Model 2 adjusts for the variables of model 1 and for gender, prevalent CVD and diabetes mellitus. Model 3 adjusts for variables of model 2 and for statin intake.

### Replication of the analysis in the LURIC cohort

**Table 4** depicts the baseline characteristics of LURIC participants stratified by tertiles of PCSK9.

**Table 4:** Baseline characteristics of LURIC participants stratified by PCSK9 tertiles.

	Total Cohort	1^st^ Tertile	2^nd^ Tertile	3^rd^ Tertile	p-value
	N = 1450	N = 498	N = 470	N = 482	
**Gender (men)**	925 (64%)	362 (73%)	295 (63%)	268 (56%)	**< 0.001**
**Prevalent CVD (yes)**	994 (69%)	312 (63%)	338 (72%)	344 (71%)	**0.002**
**Smoking (yes)**	206 (14%)	76 (15%)	69 (15%)	61 (13%)	**0.043**
**Diabetes (yes)**	637 (44%)	206 (41%)	201 (43%)	230 (48%)	0.111
**Age (years)**	67.0 [59.5–72.5]	66.4 [58.6–71.7]	67.4 [60.0–72.9]	66.9 [59.9–72.5]	0.105
**BMI (kg/m**^**2**^**)**	27.0 [24.7–29.7]	26.7 [24.7–29.4]	27.0 [24.8–29.9]	27.4 [24.6–29.8]	0.497
**Systolic BP (mmHg)**	143 [127–159]	143 [126–159]	144 [129–158]	142 [126–161]	0.708
**Diastolic BP (mmHg)**	81 [73–88]	82 [74–90]	80 [73–89]	81 [73–87]	0.302
**GFR (ml/min/1.73 m**^**2**^**)**	74.7 [65.0–81.3]	76.5 [65.0–82.3]	73.5 [64.9–80.5]	74.8 [65.1–81.0]	0.155
**PCSK9 (ng/ml)**	208 [161–264]	146 [123–163]	208 [193–223]	291 [264–334]	***< 0*.*001***
**Total Cholesterol (mg/dl) (mg/dl)**	189 [165–215]	188 [166–215]	190 [165–216]	189 [163–215]	0.646
**HDL Cholesterol (mg/dl)**	37 [31–45]	37 [31–44]	38 [31–45]	38 [31–45]	0.171
**LDL Cholesterol (mg/dl)**	112 [91–135]	113 [93–135]	112 [89–135]	110 [89–135]	0.833
**Triglycerides (mg/dl)**	150 [112–202]	140 [109–192]	149 [110–196]	159 [117–214]	***0*.*001***
**Apo A-I (mg/dl)**	127 [113–144]	124 [111–141]	127 [113–147]	129 [114–146]	***0*.*008***
**Apo B (mg/dl)**	103 [87–118]	102 [86–117]	103 [88–118]	103 [87–120]	0.278
**hsCRP (mg/l)**	3.7 [1.5–9.0]	3.7 [1.5–8.4]	3.7 [1.3–9.1]	3.7 [1.5–9.6]	0.501
**Statin (yes)**	721 (50%)	164 (33%)	235 (50%)	322 (67%)	**< 0.001**

**CVD:** cardiovascular disease; **BMI:** body mass index; **BP:** blood pressure; **GFR:** glomerular filtration rate; **PCSK9:** proprotein convertase subtilisin/kexin type 9; **Apo A-I, B:** apolipoprotein A, B; **hsCRP:** high-sensitivity C-reactive protein. Data are presented as numbers (percentages) or median and interquartile range as appropriate. P-Values in italics are significant by Jonckheere-Terpstra test for trend.

**Fig 4:**
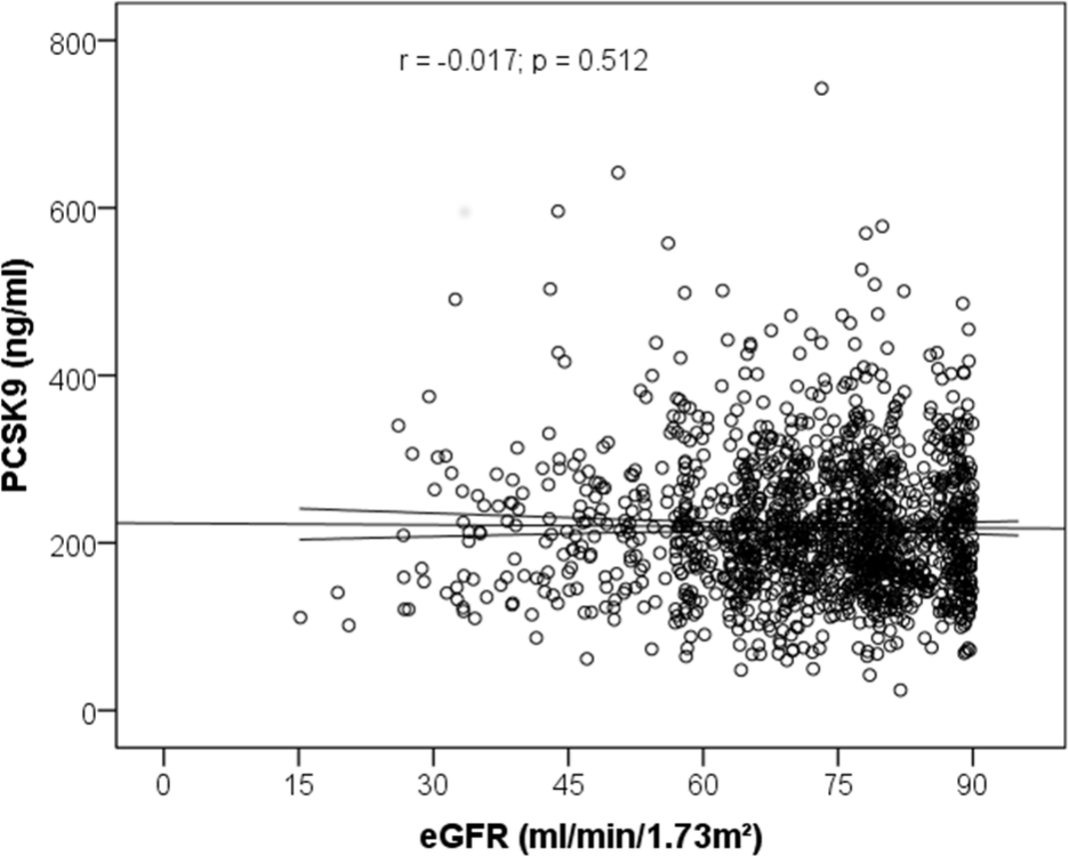
Correlation between PCSK9 and estimated glomerular filtration rate (GFR) assessed by Spearman test in LURIC participants.

Among all 1450 patients analysed, PCSK9 levels correlated negligibly with Apo A-I (r = 0.092; p = <0.001), triglycerides (r = 0.084; p <0.001) and with HDL cholesterol (r = 0.061; p = 0.021) but not with total cholesterol (r = 0.006; p = 0.832), LDL cholesterol (r = -0.018; p = 0.489), Apo B (r = 0.026; p = 0.321) or GFR (r = -0.017; p = 0.512; **Fig 4**).

After stratification of the cohort by intake of statins, only weak associations between PCSK9 and total cholesterol (r = 0.106; p = 0.004), LDL cholesterol (r = 0.100; p = 0.007), Apo B (r = 0.107; p = 0.004), HDL cholesterol (r = 0.102; p = 0.006), Apo A-I (r = 0.126; p = 0.001) and triglycerides (r = 0.094; p = 0.011) were seen in patients (n = 729) not on statins. However, no correlation between PCSK9 and GFR (r = 0.013; p = 0.733) was detectable in those patients not on statins.

In statin users (n = 721) no correlation between PCSK9 and total cholesterol (r = 0.058; p = 0.119), LDL cholesterol (r = 0.028; p = 0.448), Apo B (r = 0.046; p = 0.215), triglycerides (r = 0.041; p = 0.273) or GFR (r = -0.024; p = 0.528) was seen; only a weak association appeared between PCSK9 and HDL cholesterol (r = 0.093; p = 0.012) and Apo A (r = 0.142; p < 0.001), respectively.

**Fig 5:**
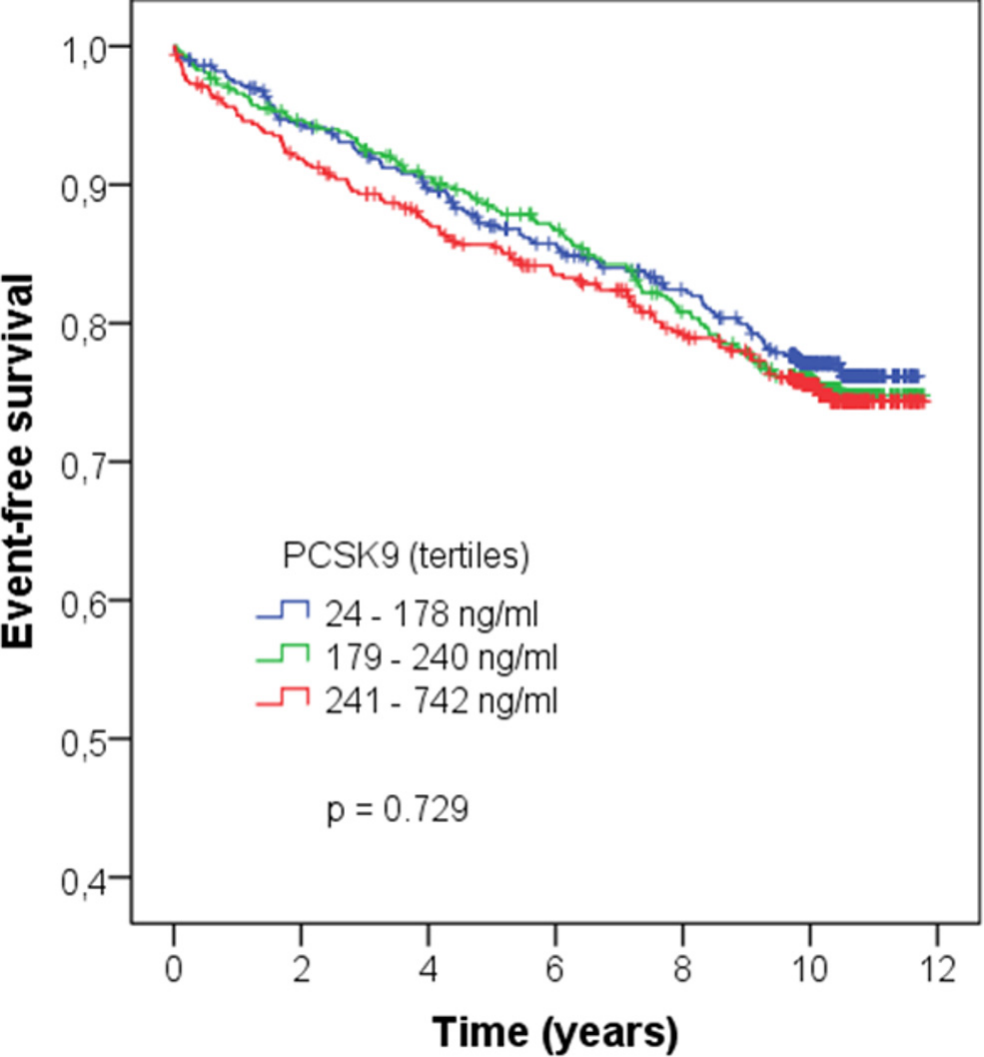
Association of PCSK9 tertiles with cardiovascular death in LURIC participants; Kaplan-Meier analysis followed by log-rank test.

During a median follow-up time of 10.0 [7.3–10.6] years 497 patients died, of whom 335 patients died from cardiovascular causes; **Table 5** summarizes clinical characteristics according to event status. In Kaplan-Meier analysis, tertiles of PCSK9 were not associated with cardiovascular deaths (p = 0.729; **Fig 5**).

**Table 5:** Baseline characteristics of LURIC stratified by cardiovascular event status.

	Total Cohort	Alive	CV Death	Non CV Death	p-value
	N = 1450	N = 953	N = 335	N = 162	
**Gender (men)**	925 (64%)	588 (62%)	222 (66%)	115 (71%)	**0.042**
**Prevalent CVD (yes)**	994 (69%)	609 (64%)	270 (81%)	115 (71%)	**<0.001**
**Smoking (yes)**	206 (14%)	151 (16%)	30 (9%)	25 (15%)	**0.001**
**Diabetes (yes)**	637 (44%)	344 (36%)	211 (63%)	82 (51%)	**<0.001**
**Age (years)**	67.0 [59.5–72.5]	64.1 [56.9–70.1]	70.8 [66.0–75.8]	72.1 [66.0–75.6]	***<0*.*001***
**BMI (kg/m**^**2**^**)**	27.0 [24.7–29.7]	27.1 [24.8–29.8]	26.7 [24.7–29.8]	26.1 [24.2–29.0]	***0*.*046***
**Systolic BP (mmHg)**	143 [127–159]	141 [124–157]	147 [130–163]	145 [131–163]	***<0*.*001***
**Diastolic BP (mmHG)**	81 [73–88]	82 [74–89]	80 [72–88]	79 [73–86]	**0.146**
**GFR (ml/min/1.73 m**^**2**^**)**	74.7 [65.0–81.3]	76.4 [67.0–81.8]	70.2 [57.9–78.8]	73.4 [62.9–81.1]	***<0*.*001***
**PCSK9 (ng/ml)**	208 [161–264]	208 [162–262]	206 [161–264]	211 [153–271]	0.999
**Total Cholesterol (mg/dl) (mg/dl)**	189 [165–215]	190 [166–217]	185 [161–213]	186 [165–212]	0.158
**HDL Cholesterol (mg/dl)**	37 [31–45]	38 [32–45]	36 [30–44]	37 [30–45]	***0*.*006***
**LDL Cholesterol (mg/dl)**	112 [91–135]	112 [90–136]	112 [90–136]	112 [91–135]	0.936
**Triglycerides (mg/dl)**	150 [112–202]	150 [113–201]	150 [107–204]	148 [113–201]	0.932
**Apo A-I (mg/dl)**	127 [113–144]	129 [114–147]	122 [107–139]	125 [113–143]	***0*.*001***
**Apo B (mg/dl)**	103 [87–118]	102 [87–118]	103 [87–119]	103 [87–118]	0.957
**hsCRP (mg/l)**	3.7 [1.5–9.0]	3.0 [1.2–8.0]	5.6 [2.2–11.4]	4.0 [2.0–10.3]	***<0*.*001***
**Statin (yes)**	721 (50%)	478 (50%)	157 (47%)	86 (53%)	0.387

**CVD:** cardiovascular (disease); **BMI:** body mass index; **BP:** blood pressure; **GFR:** glomerular filtration rate; **UAE:** urinary albumin excretion; **PCSK9:** proproteinconvertase subtilisin/kexin type 9; **Apo A-I, B:** apolipoprotein A, B; **hsCRP:** high-sensitivity C-reactive protein. Data are presented as numbers (percentages) or median and interquartile range as appropriate. P-Values in italics are significant by Jonckheere-Terpstra test for trend.

Separate analyses stratified by statin intake did not yield different results (no statin: p = 0.772; statin: p = 0.611).

In multivariate Cox-Regression analysis (model adjusting for sex, presence of cardiovascular disease, smoking status, diabetes mellitus, age, BMI, systolic blood pressure, GFR, apolipoprotein A-I and C-reactive protein) male sex (p = 0.020; HR 1.68, 95% CI: [1.024–1.332]), no prevalent cardiovascular disease (p = 0.002; HR 0.830, 95% CI: [0.736–0.936]), previous smoking (p = 0.019; HR 1.162, 95% CI [1.025–1.317]), diabetes mellitus (p <0.001; HR 1.425, 95% CI: [1.278–1.589], age per year (p < 0.001; HR 1.024, 95% CI: [1.017–1.030]) and GFR per 1ml/min/1.73m^2^ (p < 0.001; HR 0.987, 95%CI: [0.982–0.991]) predicted cardiovascular death.

Taken together, results from the independent LURIC study fully support the results obtained in the CARE FOR HOMe study.

## Discussion

The two main findings obtained in the two study cohorts are that the degree of renal function impairment has no effect on total PCSK9 plasma concentrations. In addition, PCSK9 levels did not predict cardiovascular outcomes in patients with GFR between 15 and 90 ml/min/1.73m^2^.

To the best of our knowledge this is by far the largest analysis of PCSK9 plasma concentrations in patients with decreased GFR accompanied by validation in an independent cohort.

In cross-sectional analyses, PCSK9 was associated with BMI, total cholesterol, Apo B, Apo A-I and triglycerides in the overall CARE FOR HOMe cohort, which is in line with findings from the community-based Dallas Heart Study [20]. Statins increase PCSK9 levels by activating the sterol regulatory element-binding protein-2 (SREBP2), which in turn increases expression of PCSK9 [11,21]. Therefore, patients not on statins and statin-users were assessed separately. In these analyses, the associations between PCSK9 and other lipid parameters remained robust. As expected, we found higher PCSK9 levels among statin users compared to non-statin users. Findings obtained from LURIC were consistent. We therefore conclude that kidney function, assessed as GFR, has no major impact on PCSK9 concentrations.

The lack of an association between PCSK9 levels with CV outcome in patients with decreased GFR differs from findings in the general population where genetically determined lower PCSK9 levels are associated with lower LDL-C levels and consecutively lower CV risk in the Atherosclerosis in Risk in Communities study (ARIC) [22]. Similarly, PCSK9 correlates with CV risk in patients with stable coronary artery disease [23]. Of note, the ARIC study enrolled a study population in the setting of primary prevention and no information on kidney function was given in this analysis [22], whereas in the cohort of patients with stable coronary artery disease CKD was defined as an exclusion criterion [23]. In contrast, the CARE FOR HOMe study represents a referred CKD patient population with 30% of patients having prevalent CV disease. Therefore differences in study design and study populations might account for these different findings, since patients with CKD are characterized by specific metabolic and lipidologic alterations.

Of note, proteinuria is a further important modifier of uremic dyslipidemia leading to a distinct dyslipidemia in CKD patients characterised by increased total- and LDL cholesterol [7]. Two previous reports in smaller groups (n = 15 Korean patients with nephrotic syndrome, i.e. proteinuria >3.5g/d, and n = 39 patients from the Netherlands with protein excretion of 1.9 [0.9–3.3] g/day) of patients with marked proteinuria observed rises in PCSK9 [24,25]. We did not find a correlation between urinary albumin excretion and PCSK9 levels. Of note, the level of urinary albumin excretion was only in the range of microalbuminuria in CARE FOR HOMe (median: 35, IQR [8–193] mg/g creatinine) thus making it difficult to compare these two studies with CARE FOR HOMe. These studies are complemented by a third study in 66 haemodialysis patients in whom PCSK9 concentrations were measured and compared to non-CKD patients [26]. Approximately half of the haemodialysis patients were taking statins; the overall haemodialysis cohort had significantly lower LDL-C levels and lower PCSK9 concentrations compared to non-CKD patients, however when stratifying haemodialysis patients according to statin intake, PCSK9 concentrations between haemodialysis patients on statins and non-CKD patients did not differ significantly.

Taken together, based on the lack of an effect of GFR on PCSK9 in CARE FOR HOMe and LURIC we would expect equal LDL-C lowering in CKD compared to non-CKD populations by pharmacologic inhibition of PCSK9.

### Study strengths and limitations

Statin intake in nearly half of the study participants in CARE FOR HOMe and LURIC could be regarded as a limitation, since statins modify PCSK9 concentrations. However, analyses stratified according to statin intake did not materially change the results. More importantly, this high prevalence of statin intake represents current medical practice in a real life setting in which anti-PCSK9 agents potentially would be used.

Outcome definitions in CARE FOR HOMe and LURIC differed because the studies were performed independently, which is a potential limitation of our analysis. As we present data from cohort studies we are not able to draw a conclusion on potential mechanisms and causality.

A particular strength of our study is complete follow-up in both studies and validation in a second independent cohort of LURIC participants with GFR between 15 and 90 ml/min/1.73 m^2^. Obviously, the question whether inhibition of PCSK9 is able to reduce CV events in patients with impaired renal function cannot be answered from our data.

## Conclusions

In conclusion, our study confirms that patients with decreased GFR present with specific lipid abnormalities that differ from other secondary prevention populations. Since renal impairment can predispose to statin side effects and increased risk of statin intolerance [27], alternative LDL lowering drugs might be useful for patients with decreased GFR.

Whether PCSK9 inhibitors represent such an alternative should be prospectively tested.
